# The relationship between burnout, sense of coherence and job safety attitudes among nurses after coronavirus disease 2019 in China: a cross-sectional survey

**DOI:** 10.3389/fpubh.2025.1516744

**Published:** 2025-02-19

**Authors:** Fang Xue, Jing Liu, Tong Zhou, Xiuchuan Li, Chunfang Liu, Xueer Li, Shuoshuo Li, Ping Ye, Jing Zhang

**Affiliations:** ^1^School of Nursing, Bengbu Medical University, Bengbu, China; ^2^Department of Oncology, First Affiliated Hospital of Bengbu Medical University, Bengbu, China; ^3^Department of Cardiovascular Medicine, First Affiliated Hospital of Bengbu Medical University, Bengbu, China; ^4^Nursing Department, First Affiliated Hospital of Bengbu Medical University, Bengbu, China; ^5^Department of Gynecologic Oncology, First Affiliated Hospital of Bengbu Medical University, Bengbu, China; ^6^School of Mental Health, Bengbu Medical University, Bengbu, China

**Keywords:** COVID-19, nurse, burnout, sense of coherence, safety attitude

## Abstract

**Objective:**

To investigate burnout, sense of coherence (SOC), and job safety attitudes among nurses in China after the coronavirus disease 2019 (COVID-19) pandemic.

**Methods:**

This cross-sectional study included 302 front-line nurses from Fangcang shelter hospitals (FSHs) in China. Descriptive, univariate, Pearson’s correlation, and multiple linear regression analyses were conducted to analyze factors related to job safety attitudes of nurses toward FSHs.

**Results:**

The incidence of burnout among nurses in FSHs was 65.2%; 57.9% had a low level of SOC, the score for safety attitude was 74.47 (standard deviation = 13.33), and the agreement rate was 51%. Burnout was negatively correlated with SOC (*r* = −0.399, *p* < 0.01) and safety attitudes (*r* = −0.141, *p* < 0.05), and SOC was positively correlated with safety attitudes (*r* = 0.428, *p* < 0.01). Personal accomplishment, depersonalization, changes in working hours, manageability, and marital status explained 33.8% of the variance in work safety attitudes.

**Conclusion:**

This study investigated the levels of burnout, SOC, and safety attitudes among nurses after COVID-19 in Chinese FSHs for the first time, and analyzed the associated factors. The results are valuable for improving the quality of nursing safety as well as patient safety management in FSHs.

## 1 Introduction

The unpredictable behavior of severe acute respiratory syndrome coronavirus-2 (SARS-CoV-2) and insufficient national responses have contributed to the continued global pandemic of coronavirus disease 2019 (COVID-19). There were more than 633,000,000 confirmed COVID-19 patients and more than 6,596,000 deaths worldwide by November 2022 (1). Global medical and care resources face enormous challenges, and the International Health Regulations Emergency Committee has expressed concerns regarding the heavy workload and associated burnout experienced by healthcare workers worldwide (2). China has adopted active response strategies since the outbreak of COVID-19 in Wuhan. Under the normalized management of the pandemic, China adhered to the general strategy of “external prevention of import, internal prevention of rebound” and the policy of “dynamic COVID-zero” (3), which brought the pandemic under control. The mortality rate and number of severe cases have continued to decline, and Fangcang shelter hospitals (FSHs) have been an important part of the pandemic response.

FSHs are strategies that were implemented for the first time in China to manage COVID-19. These large temporary hospitals expanding and modifying the medical services of existing public facilities in the cities (4). Patients with mild COVID-19 and asymptomatic individuals were treated at FSHs; they not only provide appropriate medical care but also monitor the disease, implement strategic classification, and provide timely transfer of patients with progressive disease to designated hospitals (5), reducing the pressure on traditional hospitals. A certain number of staff members are required to operate an FSH. As the number of infected people has grown rapidly, and there is often a shortage of local medical staff, China has actively mobilized national support through measures such as pulling widespread healthcare staff to help Wuhan, Hong Kong, Shanghai, and other areas. The efforts and dedication of Chinese nurses have been instrumental in the containment of COVID-19 (6).

Nurses are the main forces behind FSHs; they enter these hospitals with protection while working, and live in isolation in a unified living area after work. Although majority of the patients in FSHs have mild cases, nurses also face many challenges such as an increased workload as a result of the large number of patients they have to manage (7), the possibility of infection due to direct contact with confirmed patients (8, 9), and the fear for parents and children due to being away from home (9, 10). Epidemic outbreaks and immense stress have had significant psychological impact on healthcare workers (11). One study (12) found that the proportion of front-line nurses who experienced anxiety, depression, and insomnia was significantly higher during the outbreak and that nurses in FSHs were more likely to experience psychological problems than other front-line nurses during the stable phase of the epidemic. These adverse influences may lead to job burnout among front-line nurses (7, 9). Burnout syndrome is an employee’s emotional response to chronic work stress that manifests as depersonalization and reduced professional efficacy (13). Healthcare workers are at a high risk of burnout (14–16), which is closely related to quality of care and patient safety (11, 14). Sense of coherence (SOC), a core concept of the salutogenic model of health proposed by Antonovsky, is a stable psychological tendency in individuals (17). When people experience stressful events, SOC shapes their overall feelings and perceptions about life, reflecting on their outlook on life and their ability to cope with stress (18). Several studies (19–22) have demonstrated that SOC helps people successfully cope with stressful events. Previous studies (23, 24) have shown that healthcare workers were at a higher risk of psychological problems during SARS and H1N1. When nurses have high levels of SOC, they face difficult situations with a positive mindset, mobilize their resources to cope with stress, and have reduced negative emotions and health risks (25–27). Patient safety has always been a primary goal of global health (28). It is defined as the prevention and avoidance of risk, harm, or adverse events during healthcare delivery (29). Safety culture is the product of the values, attitudes, perceptions, abilities, and behavioral patterns of individuals and groups, which determine the commitment, style, and proficiency of an organization’s health and safety management (30). The safety attitudes of healthcare staff towards patients are an important part of the safety culture of hospitals. As surveys cannot measure all aspects of culture, the safety climate of a healthcare organization should be assessed to determine its strengths and weaknesses (31). The COVID-19 pandemic is not only related to physical health, but also to mental health, sleep problems, depression, burnout and traumatic stress. A systematic evaluation and meta-analysis of 85 studies shows that nurse burnout is negatively correlated with patient safety, patient satisfaction, and nursing quality; Secondly, during the COVID-19 pandemic, the prevalence of nurses’ job burnout was even higher due to hospital overcrowding and understaffing (32).

During the COVID-19 pandemic, the risk to nurses’ mental health increased rapidly. The COVID-19 pandemic is not only related to physical health, but also to mental health, sleep problems, depression, burnout, and traumatic stress. Healthcare workers are at a high risk of burnout, which is closely related to quality of care and patient safety. We conducted a study on the current status and correlation of post-traumatic stress fatigue, coherence, and work safety attitudes among Chinese nurses after COVID-19 in 2019. A literature search revealed that published evidence has focused on determining the level of stress in HCWs in the context of the COVID-19 pandemic. However, there is less research on the relationship between Burnout, Sense of Coherence and Job Safety Attitudes among nurses experiencing frontline work on the outbreak after re-entering the workforce following the New Crown Pneumonia pandemic. Therefore, we were the first to take on the challenge of assessing those factors among nurses. The aim of this study was to investigate the levels of burnout, SOC, and safety attitudes among FSH nurses after the pandemic period of neocoronavirus pneumonia and to analyse the factors related to their attitudes towards safe work. The research results have important reference value for improving the nursing quality and patient safety management of frontline nurses who have experienced FSH during their return to work.

## 2 Materials and methods

### 2.1 Study design

This study used a cross-sectional, descriptive research design and followed the STROBE statement specification for cross-sectional studies (33).

### 2.2 Setting

This study was conducted at the Anhui FSH, located on Chongming Island, Shanghai, China. All admitted patients were mildly ill with confirmed COVID-19 and asymptomatic infection, and all nurses providing healthcare services were from the Anhui Province.

### 2.3 Participants

In this study, 360 nurses who met these requirements were recruited between May 8 and 15, 2024. Due to the pandemic, the questionnaire was set up online through the Wenjuanxing app^1^ and distributed through the WeChat platform, which is the most used social networking software in China. All participants anonymously submitted their questionnaires and informed consent forms online. The inclusion criteria were officially registered nurses working in an FSH for 1 month or more who could use their smartphones normally and complete the questionnaire. The exclusion criteria were those who took an overly short time to complete the questionnaire (within 300 s), invalid questionnaires that were not logical, and incomplete or identical responses.

### 2.4 Instruments

The socio-demographic data collection form was used to collect the personal information from the participants, including gender, age, education status, marital status, if they were an only-child, having children, type of hospital, clinical rank, night shift participation, change in working hours, change in sleep time, experience with the pandemic, experience with negative life events within the last month, and level of concern about the pandemic.

The Maslach Burnout Inventory-Human Services Survey (MBI-HSS) was used to investigate burnout among nurses. The MBI-HSS is applicable to social service workers and is now widely used in burnout studies of healthcare workers (34–37). It has 22 items in three subscales: emotional exhaustion (EE), depersonalization (DP), and personal accomplishment (PA) (38). Each item is evaluated on a 7-point Likert scale ranging from 0 (never) to 6 (every day). The Chinese version of the MBI-HSS was used in the present study. The Cronbach’s *α* coefficient was previously reported to be 0.737 for the overall scale and 0.858, 0.761, and 0.757 for the subscales, respectively (39). In this study, the total Cronbach’s α coefficient was 0.845, and those of the subscales were 0.894, 0.904, and 0.874, respectively. According to the burnout norms of Chinese nurses (40), the critical value of burnout was determined as follows: EE ≤ 17, DP ≤ 3, and PA ≥ 33 were classified as mild; EE of 18–26, DP of 4–7, and PA of 25–32 were classified as moderate; and EE ≥ 27, DP ≥ 8 and PA ≤ 24 were classified as severe. For the diagnosis of burnout, some studies have used strict criteria to consider high EE, high DP, and low PA (41), while others have used loose criteria to consider high EE with high DP or low PA (42). In this study, with reference to Li’s (43) study on the Chinese population, the diagnostic criteria for burnout were as follows: (1) none: scores on all three subscales were lower than the critical value; (2) mild: scores on one subscale were higher than the critical value; (3) moderate: scores on two subscales were higher than the critical value; and (4) high: scores on all three subscales were higher than the critical value. The most recent version of official manual of MBI recommends not coding burnout as categorical. This study classified coding burnout as categorical mainly based on the odds ratios more interpretable for clinical settings.

The Sense of Coherence Scale (SOC-13), a 13-item scale developed by Antonovsky, was used to measure the SOC of the nurses (17, 44). The scale contains 13 items across three dimensions: comprehensibility, manageability, and meaningfulness. Comprehensibility is a cognitive dimension that represents feelings about stressful internal and external events; a higher score indicates that people consider stressful events to be reasonable and understandable, and that things they understand are easier to manage. Manageability is a behavioral dimension representing the degree to which people think that internal and external resources are at their disposal; a higher score indicated that people thought they had more resources at their disposal, they were more willing to solve problems that caused stress and find meaning in managing stressful events. Meaningfulness is a motivational dimension, representing the degree to which people think life is meaningful; a higher score indicates that people are more willing to devote energy to solving problems and taking responsibility. The SOC-13 is evaluated on a 7-point Likert scale from 1 (never) to 7 (often), with total scale scores ranging from 13 to 91 and higher scores representing higher levels of SOC. A total scale score of 13–63 represents a low level of SOC, 64–79 represents a moderate level, and 80–91 represents a high level (45). The Chinese version of the SOC-13 was used in this study, with a Cronbach’s alpha coefficient of 0.76 (46, 47). In this study, the overall Cronbach’s alpha coefficient was 0.887.

The Safety Attitude Questionnaire (SAQ) developed by Sexton et al. (30) was used to assess nurses’ perceptions of safety attitudes in their work environments (30). The generic SAQ Short-Form version contains 36 items across six dimensions: teamwork climate, safety climate, perceptions of management, job satisfaction, working conditions, and stress recognition. Each item is evaluated on a 5-point Likert scale ranging from 1 (strongly disagree) to 5 (strongly agree). To facilitate interpretation of the scores, the 5-point scale is converted to a 100-point scale (48, 49): scale score for a respondent = [(Mean of the items) − 1] * 25 with ≤ 50 indicating a need for improvement, and ≥ 75 indicating a positive attitude towards safety. A representative response rate of at least 60% is considered a satisfactory level of safety culture (50). The Chinese version of the SAQ (51) was used in this study, with a total Cronbach’s *α* coefficient of 0.945; the subscales have Cronbach’s α coefficients of 0.785–0.899. The overall Cronbach’s alpha in this study was 0.926, and the Cronbach’s alpha for the subscales were 0.749–0.914.

### 2.5 Study size

The study size was calculated according to the formula

$$n=\frac{{Z^{2}}_{1-\frac{\alpha }{2}}p\left(1-p\right)}{d^{2}}$$

where p is the presenting rate, d is the tolerance error, and Z_1−*α*/2_ is the statistic for the significance test. According to a study of burnout among Chinese nurses, the current rate of burnout was 64.5% (16), the allowable error was 0.1p, and Z_1−α/2_ was 1.96. Considering that 20% of the questionnaires were expected to be invalid, at least 254 questionnaires were distributed.

### 2.6 Statistical methods

SPSS software version 25.0 (SPSS-IBM Corporation, New York, NY, United States) was used for statistical analysis. Descriptive analyses were performed using means, standard deviations, frequencies, and percentages. P-P and Q-Q plots are used to evaluate the normality of data. Univariate analysis uses significance level defined as two-sided *p* < 0.05 by *t*-test and analysis of variance. A Pearson’s correlation analysis was performed for the scales and each dimension. Statistically significant factors (*p* < 0.05) were included as independent variables and SAQ scores were used as dependent variables in a multiple linear regression analysis (stepwise) to analyze the factors that influenced the safety attitudes of nurses toward FSHs. A collinearity diagnosis was made before the analysis to ensure that there was no collinearity between variables.

### 2.7 Ethics

This study was approved by the Ethics Committee of the First Affiliated Hospital of Bengbu Medical College (approval number: BYYFY-2020KY01). Subjects were informed of the purpose, completion method, and precautions of the questionnaire in advance and the questionnaire was completed online after consent was obtained. The questionnaire was distributed in the WeChat group in the form of a QR code, and only those who voluntarily wished to complete the survey could scan the code and select “agree” to complete the questionnaire. If a participant wanted to terminate the survey at any time process, they could simply exit the interface. All questionnaires were completed anonymously, and the data were stored safely and used only for this study.

## 3 Results

**Figure 1:**
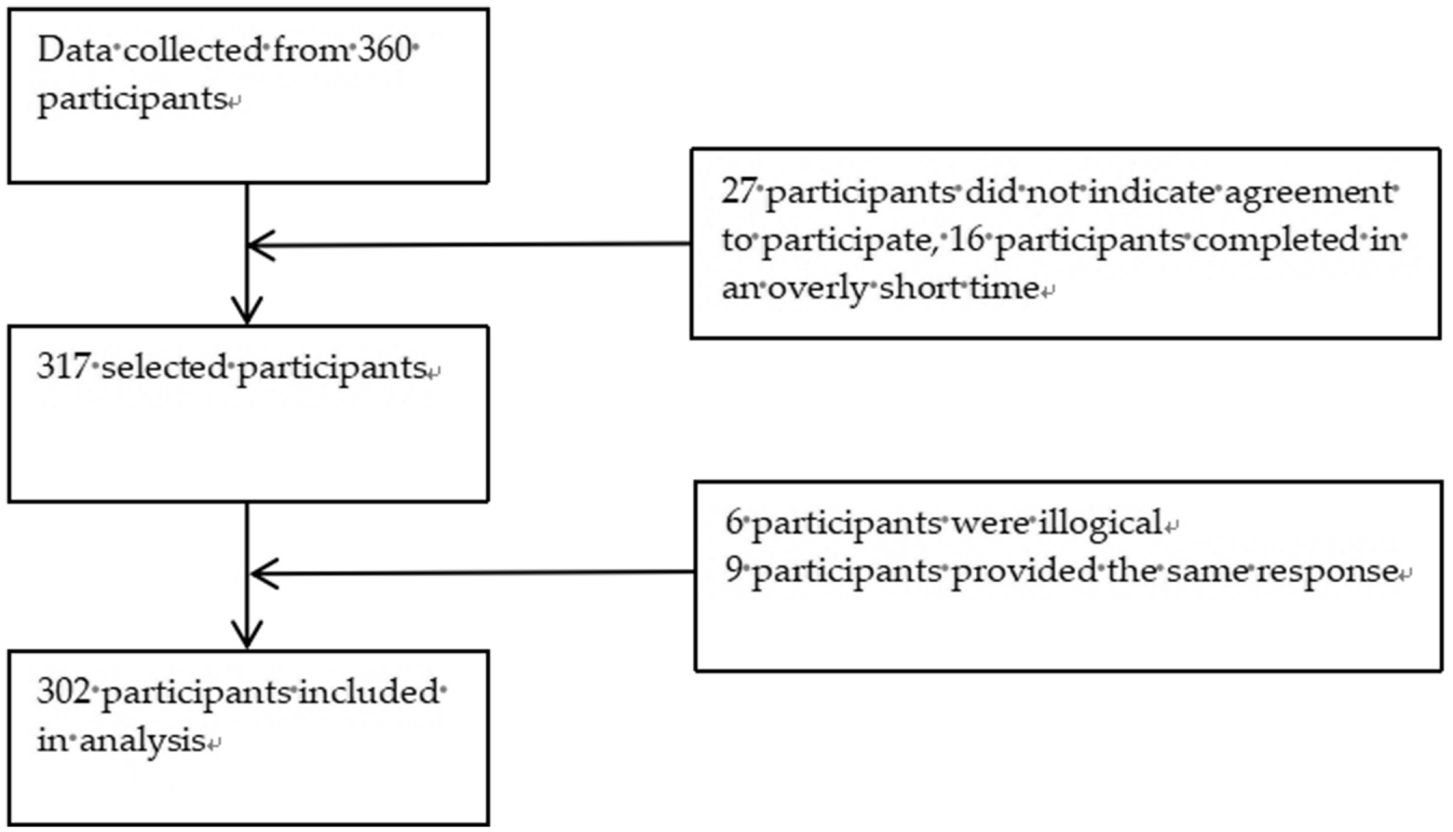
Flow chart for participants’ inclusion.

All participants were invited to participate in the survey when the QR codes were distributed in the WeChat group. A total of 360 questionnaires were distributed and 302 valid questionnaires were returned (return rate: 83.89%) (Figure 1). The researcher examined each returned questionnaire and excluded invalid questionnaires, including those that did not indicate agreement to participate (*n* = 27), were completed in an overly short time (*n* = 16), were illogical (*n* = 6), or were answered with the same response (*n* = 9). We set the questionnaire items as mandatory; if the participants missed items, the software automatically reminded them to complete these before they could submit the questionnaire to minimize possible bias in the self-reported results.

### 3.1 Sociodemographic and work characteristics

Of the 302 participants, 89.7% were female, 77.5% had a bachelor’s degree, 63.3% were married, 18.2% were only child, and 61.9% had children. The sociodemographic and work characteristics of the nurses are described in Table 1.

**Table 1:** Sociodemographic and work characteristics variables (*N* = 302).

Variables	*N* (%)
Gender	
Male	31 (10.3)
Female	271 (89.7)
Age	
≤ 30	117 (38.7)
31–40	150 (49.7)
> 40	35 (11.6)
Education status	
Associate degree	59 (19.5)
Undergraduate degree	234 (77.5)
Postgraduate	9 (3.0)
Marital status	
Single	104 (34.4)
Married	191 (63.3)
Separated/divorced/widowed	7 (2.3)
Only-child family	
Yes	55 (18.2)
No	247 (81.8)
Have children	
Yes	187 (61.9)
No	115 (38.1)
Type of hospital	
Provincial hospital	112 (37.1)
Municipal hospital	133 (44.0)
County hospital	42 (13.9)
Military hospital	15 (5.0)
Clinical rank	
Nurse	59 (19.5)
Junior nursing lead	99 (32.8)
Principal nursing lead	135 (44.7)
(Assistant) nursing manager	9 (3.0)
Participate in the night shift	
Yes	270 (89.4)
No	32 (10.6)
Change in working hours	
Increased	21 (7.0)
No change	86 (28.5)
Reduced	195 (64.5)
Change in sleep time	
Increased	18 (6.0)
No change	130 (43.0)
Reduced	154 (51.0)
Experience with pandemic	
Yes	116 (38.4)
No	186 (61.6)
Experienced negative life events within the last month	
Yes	71 (23.5)
No	231 (76.5)
Level of concern about COVID-19	
Never	19 (6.3)
Little	236 (78.1)
Worried	36 (11.9)
Very	11 (3.7)

### 3.2 Burnout, sense of coherence, and safety attitudes of nurses in Fangcang shelter hospitals

The average score of burnout among nurses in FSHs was 65.41 [standard deviation (SD) = 15.92], with an incidence of 65.2% (Table 2). The SOC score was 61.13 (SD = 13.00), with 57.9% at a low level. Table 3 shows that the average job safety attitude score of nurses was 74.47 (SD = 13.33), with a percentage agreement rate of 51.0%.

**Table 2:** Burnout and SOC scores of nurses in Fangcang shelter hospitals (*N* = 302).

Variables	Scores	None	Mild	Moderate	High
	Mean ± SD	*N* (%)	*N* (%)	*N* (%)	*N* (%)
SOC	61.13 ± 13.00	–	175 (57.9)	103 (34.2)	24 (7.9)
Comprehensibility	19.31 ± 4.19	–	–	–	–
Manageability	23.20 ± 5.93	–	–	–	–
Meaningfulness	18.62 ± 4.21	–	–	–	–
Burnout	65.41 ± 15.92	105 (34.8)	67 (22.2)	98 (32.4)	32 (10.6)
Emotional exhaustion	24.59 ± 10.10	–	75 (24.8)	99 (32.8)	128 (42.4)
Depersonalization	9.16 ± 6.56	–	70 (23.2)	73 (24.2)	159 (52.6)
Personal accomplishment	31.67 ± 8.31	–	134 (44.4)	97 (32. 1)	71 (23.5)

SD, standard deviation; SOC, sense of coherence.

**Table 3:** SAQ scores of nurses in Fangcang shelter hospitals (*N* = 302).

Items	Mean ± SD	Percentage agreement rate (%)
Teamwork climate	75.34 ± 16.10	55.3^b^
Safety climate	76.15 ± 16.22	57.6^b^
Perceptions of management	80.90 ± 17.00	55.6^b^
Job satisfaction	78.68 ± 19.40	66.9^a^
Working conditions	74.01 ± 18.92	59.6^b^
Stress recognition	61.73 ± 26.04	42.4^b^
SAQ total	74.47 ± 13.33	51.0^b^

SD, standard deviation.

a Signifies the percentage agreement rate is higher than 60%.

b Signifies the percentage agreement rate is lower than 60%.

### 3.3 Factors associated with burnout, sense of coherence, and safety attitude

The univariate analysis showed that participating in the night shift (*p* = 0.003) and experiencing negative life events within the last month (*p* = 0.003) were associated with burnout (Table 4). Age (*p* = 0.023), marital status (*p* = 0.018), having children (*p* = 0.011), type of hospital (*p* = 0.016), clinical rank (*p* = 0.032), participation in night shifts (*p* < 0.001), changes in sleep time (*p* < 0.001), negative life events within the last month (*p* < 0.001), and level of concern about the pandemic (*p* = 0.001) were all related to SOC. Age (*p* = 0.002), educational status (*p* = 0.006), marital status (*p* = 0.011), having children (*p* = 0.012), type of hospital (*p* = 0.006), clinical rank (*p* = 0.003), change in working hours (*p* = 0.006), experience with the pandemic (*p* = 0.043), and negative life events within the last month (*p* = 0.001) were related to safety attitudes.

**Table 4:** Burnout, SOC, and SAQ scores of nurses with different characteristics (*N* = 302).

Variables	Burnout	SOC	SAQ
	Mean ± SD	Mean ± SD	Mean ± SD
Sex			
Male	68.84 ± 12.44	57.74 ± 12.83	71.73 ± 14.78
Female	65.02 ± 16.24	61.51 ± 12.96	74.78 ± 13.15
*t* value	1.266	−1.548	−1.209
*p* value	0.207	0.126	0.227
Age			
≤ 30	66.28 ± 16.26	58.76 ± 12.37	71.77 ± 13.11
31–40	64.63 ± 14.18	62.17 ± 13.53	75.12 ± 13.65
41–50	65.86 ± 21.32	64.63 ± 11.38	80.66 ± 10.20
*F* value	0.366	3.817	6.591
*P* value	0.694	0.023	0.002
Education status			
Associate degree	65.19 ± 20.43	57.68 ± 11.20	71.26 ± 12.29
Undergraduate degree	65.45 ± 14.61	62.09 ± 13.33	75.65 ± 13.22
Postgraduate	65.89 ± 17.17	58.67 ± 11.52	64.78 ± 16.82
*F* value	0.011	2.925	5.136
*P* value	0.989	0.055	0.006
Marital status			
Single	66.13 ± 16.75	58.38 ± 13.57	71.39 ± 12.40
Married	64.97 ± 15.51	62.74 ± 12.27	75.94 ± 13.72
Separated/divorced/widowed	66.86 ± 16.26	58.00 ± 17.27	79.82 ± 7.22
*F* value	0.209	4.094	4.606
*P* value	0.812	0.018	0.011
Only-child family			
Yes	67.38 ± 15.32	60.15 ± 15.18	73.35 ± 16.69
No	64.98 ± 16.05	61.34 ± 12.46	74.71 ± 12.49
*t* value	1.014	−0.619	−0.57
*P* value	0.312	0.537	0.571
Have children			
Yes	64.28 ± 14.92	62.62 ± 12.39	75.98 ± 12.90
No	67.26 ± 17.33	58.70 ± 13.60	72.01 ± 13.71
*t* value	−1.529	2.566	2.538
*P* value	0.128	0.011	0.012
Type of hospital			
Provincial hospital	65.62 ± 17.88	63.12 ± 13.45	76.72 ± 12.51
Municipal hospital	66.68 ± 15.58	59.02 ± 12.48	71.94 ± 14.24
County hospital	64.05 ± 11.66	60.12 ± 12.07	73.95 ± 11.80
Military hospital	56.47 ± 11.05	67.73 ± 12.99	81.45 ± 10.38
*F* value	1.99	3.507	4.179
*P* value	0.116	0.016	0.006
Clinical rank			
Nurse	65.25 ± 20.76	58.25 ± 11.78	72.61 ± 14.23
Junior nursing lead	66.69 ± 12.57	60.18 ± 13.60	72.24 ± 13.36
Principal nursing lead	63.80 ± 13.46	62.51 ± 12.75	76.09 ± 12.55
(Assistant) nursing manager	76.67 ± 35.17	69.56 ± 12.71	86.69 ± 9.76
*F* value	2.2	2.972	4.658
*P* value	0.088	0.032	0.003
Participate in the night shift			
Yes	64.47 ± 15.75	62.23 ± 12.79	74.86 ± 13.19
No	73.41 ± 15.31	51.78 ± 10.75	71.19 ± 14.30
*t* value	−3.044	4.439	1.474
*P* value	0.003	< 0.001	0.141
Change in working hours			
Increased	63.90 ± 14.70	60.62 ± 11.63	65.65 ± 12.75
No change	66.62 ± 16.84	59.77 ± 13.35	75.65 ± 13.56
Reduced	65.05 ± 15.68	61.78 ± 12.96	74.89 ± 13.01
*F* value	0.39	0.733	5.166
*P* value	0.677	0.481	0.006
Changes in sleep time			
Increased	62.83 ± 17.22	57.39 ± 12.48	70.92 ± 13.50
No change	63.36 ± 15.59	64.67 ± 12.06	75.45 ± 12.24
Reduced	67.45 ± 15.88	58.57 ± 13.14	74.06 ± 14.16
*F* value	2.601	9.028	1.063
*P* value	0.076	< 0.001	0.347
Experience with pandemic			
Yes	65.53 ± 16.79	62.88 ± 13.078	76.43 ± 12.66
No	65.34 ± 15.40	60.03 ± 12.83	73.24 ± 13.63
*t* value	0.104	1.861	2.028
*P* value	0.917	0.064	0.043
Experienced negative life events within the last month			
Yes	70.28 ± 13.75	50.63 ± 11.39	69.73 ± 13.18
No	63.92 ± 16.27	64.35 ± 11.69	75.92 ± 13.06
*t* value	2.984	−8.7	−3.49
*P* value	0.003	< 0.001	0.001
Level of concern about this pandemic			
Never	71.74 ± 30.49	70.37 ± 15.72	80.87 ± 10.17
Little	64.80 ± 15.10	61.11 ± 12.52	74.30 ± 13.25
Worried	64.69 ± 9.68	59.03 ± 11.19	73.58 ± 12.51
Very	70.00 ± 12.53	52.45 ± 15.58	69.89 ± 19.64
*F* value	1.451	5.386	1.977
*P* value	0.228	0.001	0.117

### 3.4 Relationship between burnout, sense of coherence, and safety attitude in Fangcang shelter hospitals

There was a significant negative correlation between burnout and SOC (*r* = −0.399, *p* < 0.01), a negative correlation between burnout and safety attitude (*r* = −0.141, *p* < 0.05), and a significant positive correlation between SOC and safety attitude (*r* = 0.428, *p* < 0.01) (Table 5).

**Table 5:** Correlation coefficients between burnout, SOC, and SAQ (*r*).

	1	2	3	4	5	6	7	8	9
1. Burnout	–	−0.399**	−0.248**	−0.234**	−0. 173**	−0.207**	−0. 157**	0.247**	−0.141*
2. SOC-13		–	0.521**	0.521**	0.465**	0.496**	0.462**	−0.340**	0.428**
3. Teamwork climate			–	0.817**	0.754**	0.667**	0.591**	−0.1	0.796**
4. Safety climate				–	0.792**	0.646**	0.599**	−0.071	0.810**
5. Perceptions of management					–	0.773**	0.725**	0.025	0.892**
6. Job satisfaction						–	0.787**	−0.072	0.835**
7. Working conditions							–	−0.007	0.819**
8. Stress recognition								–	0.277**
9. SAQ total									–

**p < 0.01; *p < 0.05.

### 3.5 Multiple linear regression analysis of work safety attitude

The SAQ of nurses in FSHs was taken as the dependent variable for a stepwise multiple linear regression, with burnout, SOC, and sociodemographic variables with significant relationships with safety attitude as independent variables. The model created from the results explained 33.8% of the variance in work-safety attitudes, which was statistically significant (*F* = 31.674, *p* < 0.001). In order of importance, personal accomplishment (*β* = 0.343; *p* < 0.001), depersonalization (*β* = −0.212; *p* < 0.001), manageability (*β* = 0. 159; *p* = 0.005), change in working hours (*β* = −0. 151; *p* = 0.002) and marital status (*β* = 0.097; *p* = 0.046) predicted safety attitudes and were statistically significant. Personal accomplishment, manageability, and marital status positively predicted safety attitudes, whereas depersonalization and changes in working hours negatively predicted safety attitudes (Table 6).

**Table 6:** Multiple linear regression on nurses’ safety attitude in Fangcang shelter hospitals (*N* = 302).

Independent variables	Unstandardized coefficients		Standardized coefficients				
	*B*	*SE*	*β*	*t*	*P*	B 95% CI	VIF
(Constant)	53.853	5.012		10.745	< 0.001	43.989–63.716	–
Personal accomplishment	4.399	0.66	0.343	6.666	< 0.001	3.1–5.698	1.202
Depersonalization	−2.152	0.559	−0.212	−3.848	< 0.001	−3.253 to −1.051	1.375
Manageability	2.019	0.711	0.159	2.842	0.005	0.621–3.418	1.428
Change in working hours	−3.63	1.143	−0.151	−3.175	0.002	−5.88 to −1.38	1.033
Marital status	2.517	1.257	0.097	2.003	0.046	0.043–4.991	1.072

Dependent variable: SAQ total.

Durbin–Watson = 1.915; F = 31.674, *p* < 0.001; *R* = 0.590; *R*^2^ = 0.349; Adjusted *R*^2^ = 33.8%.

CI, confidence interval; SE, standard error; *β*, standardized regression coefficient.

## 4 Discussion

### 4.1 Burnout, sense of coherence and safety attitude of nurses in Fangcang shelter hospitals

With the extension of working hours, people who experience job burnout become emotionally exhausted, their sense of identity with regard to their own abilities and professional skills declines, and their attitudes towards work and patients become negative and indifferent (7). In this study, 42.4% of respondents reported high EE, 52.6% reported high DP, and 44.4% reported low PA. These results indicate that FSH nurses are at a high risk of burnout. Due to the special characteristics of FSHs, nurses leave their familiar working environments and live in isolation from their families, relatives, and friends, which reduces their social activities and increases their feelings of isolation (9). In our study, nurses reported higher DP, which may be related to this isolation.

In addition, while working in FSHs, nurses must shoulder excessive workloads, make direct contact with confirmed COVID-19 patients, and be concerned about becoming infected with COVID-19. During the round of the pandemic occurring when this study was conducted, the majority of patients were infected with the SARS-CoV-2 Omicron variant. This variant has characteristics of rapid transmission, the ability to escape defenses of the immune system, poor immunity to the COVID-19 vaccine, and a high risk of reinfection (52), making it more difficult to control Because of this, nurses often encountered more severe challenges during this period. These stressful events associated with COVID-19 is related to the negative emotions among nurses in FSHs (7–9), such as anxiety and depression, and may exhaust nurses’ positive emotional reserves, leading to burnout.

In our study, the SOC score for FSH nurses was higher than that of nurses in hospitals of Anhui Province (53); the “manageability” dimension having the highest score. During the 2 years since the COVID-19 pandemic, China has been exploring localized epidemic prevention policies (4, 54, 55). Every year, Chinese hospitals simulate an outbreak and conduct training on epidemic protection strategies. The Chinese government and community provide human, financial, and material support to FSHs during their operations. The high score on the “manageability” dimension may be related to the fact that daily emergency drills and good social support led nurses to believe that internal and external resources were at their disposal and that the COVID-19 outbreak was manageable, helping them find meaning in their work at the hospital. In the face of difficult and stressful events, SOC can protect individuals from damage. Kikuchi et al. (56) found a negative correlation between SOC and shiftwork, job rank, and overtime. Interestingly, in our study, burnout was higher and SOC lower among those not working night shifts. This may be because most night shift participants were front-line nurses who were mainly responsible for the care of asymptomatic and mildly infected patients; their working frequency was regular and they had sufficient protective materials and social support. Additionally, most of those who did not participate in the night shift were administrative staff responsible for the safe operation of the FSH; they needed to arrive earlier than others, participate in the design, planning, and coordination of the FSH, and undertake the training and assessment of incoming staff to ensure that the infection rate in the hospital remained low. After the opening of FSHs, it is necessary to provide good human resource management, staff and patient safety management, life care, and coordination of materials and equipment. Consequently, these factors are related to the psychological stress and burnout of managers (57, 58).

The SAQ agreement rate was below international standards (60%) (51). Job satisfaction scores were the highest among the six subscales and results were higher than those reported by nurses in general tertiary hospitals in China (59), Poland (49), Australia (60), and Malaysia (61). Job satisfaction reflects nurses’ positive evaluations of their workplaces. FSHs have become a part of a regular initiative to combat the COVID-19 pandemic in China. Although hospital conditions are not as good as those in formal hospitals, working staff still express high satisfaction with support from various sources.

The teamwork climate reflects the quality of relationships among team members as well as the collaborative climate (31). The nurses in FSHs were redeployed by the government, and although they came from different units, they were able to maintain a good teamwork climate during the fight against COVID-19. The safety climate represents the culture of safety at a particular point and measures the perceptions of healthcare personnel regarding a healthcare organization’s systems and policies for patient safety. The safety climate score of nurses in FSHs was high, likely related to the fact that managers took a series of measures to ensure the normal operation of the hospital and protect staff from infection, including optimizing the workflow, systematic knowledge training, scientific setup of shifts, establishing a collaborative model, focusing on key populations, paying attention to psychological issues, dividing management into zones, strengthening the supervision of infection control, verifying the implementation of the system, and regularly opening the clinic for staff nucleic acid testing (62). In our study, perceptions of management were positive, indicating that nurses were receptive to relevant decisions regarding staff, patients, and organizational management. Several studies (49, 61, 63, 64) have shown that nurses’ reported results on safety climates and perceptions of hospital management were lower than those found in this study. This indicates that the safety management measures adopted by the FSH managers are active.

As FSHs are innovative models, managers must solve many problems to identify the best solutions. Chinese managers usually prefer to solve problems through brainstorming, empowerment, and democracy, which means that solutions to many problems rely on the wisdom of most employees. This is conducive to finding a balance between employees and conflicts during the problem-solving process. Managers work with staff to examine safety hazards and obtain adequate resources and support, which can influence staff to develop positive attitudes towards patients (65). A study by Rasool et al. (66) indicated that occupational stress is related to safety and productivity. The stress recognition scores reported by nurses in this study were lower than those of in Sweden (64), Australia (60), Brazil (67), and tertiary hospitals in China (59). According to the conservation of resources theory, when people’s resources are depleted, they enter a defensive mode to protect themselves, usually becoming aggressive or behaving irrationally (68). During the pandemic, nurses were overworked and under stress, and had no time to reflect on their mental health (69). In situations of poor stress recognition, when nurses’ positive emotions are exhausted, it can have a detrimental related to patient safety. Therefore, better education and training on stress recognition are important to improve the safety climate of FSHs.

### 4.2 Between burnout, sense of coherence, and safety attitudes among Fangcang shelter hospital nurses

During a pandemic, the psychological health of healthcare workers is of great importance to society. High levels of stress and negative mood can exacerbate burnout among healthcare workers. High burnout is associated with a decreased quality of care and increased patient-related adverse events (70). SOC is an important factor in the management of stress and burnout, and also has a relationship with job satisfaction (25–27, 71). Pachi et al. (72) identified SOC as a negative moderator of the burnout-depression relationship, consistent with our findings. SOC can be improved by improving nurses’ social support, reducing burnout (73). Despite the severe challenges faced by countries worldwide from COVID-19, FSH managers have provided a series of measures to alleviate the tremendous stress and negative emotions of front-line nurses, such as adequate protective equipment, appropriate working hours, regular rest and sleep, standard disease protection measures, regulations and disposal procedures appropriate for FSHs, food and daily necessities, and psychological counsellors.

### 4.3 Factors related to nurses’ attitudes towards job safety attitudes in Fangcang shelter hospitals

Tan et al. (58) noted that shifts lasting ≥8 h were associated with higher burnout scores, while healthcare workers who voluntarily redeployed to field support had lower burnout levels. In this study, increased working hours were associated with lower safety attitude scores; however, no significant differences were observed in burnout or SOC scores. This may be due to the characteristics of Chinese hospitals, the uneven distribution of medical resources, the high number of patients attending provincial and municipal hospitals, high workloads, and long working hours, which have become the norm for nurses. Although there were many patients and a high workload in the FSHs, the managers set the working time at the hospital to 6 h. The reduction in total working hours and the voluntary acceptance of redeployment may equalize the difference in burnout scores caused by changes in working hours.

Consistent with the study by Moazez et al., marital status influenced perceptions of safety attitudes (74); however, the results of Magalhaes et al. (75) showed that safety attitudes were not related to marital status. We believe that marriage is a reflection of personal responsibility and that married nurses may assume more family responsibility, receive more family support, value the importance of work, and invest in it more than unmarried and divorced individuals do.

Liu et al. (70) concluded that high burnout and low job satisfaction were associated with decreased quality of care and increased patient-related adverse events. The results of a systematic evaluation and meta-analysis of 85 studies demonstrated that nurse burnout was associated with lower health care quality and safety and lower patient satisfaction (32). Multiple supportive factors allow nurses in FSHs to perceive COVID-19 as manageable and understandable and to find meaning in their work. Diverse rewards, especially nonfinancial ones, can promote nursing excellence and help ensure a high level of quality care and patient safety (76). In our study, we found that personal accomplishment and manageability positively predicted safety attitudes, while depersonalization negatively predicted them. This is easy to understand because nurses in FSHs consider it meaningful to accept a unified deployment, support Shanghai healthcare, and fight the pandemic. Additionally, global attention toward nurses is increasing, and their social status is constantly improving. The Chinese health department recognizes the contributions of nurses in FSHs and awards excellent anti-pandemic nurses, which enhances their professional identity and personal achievement. High personal accomplishment and low depersonalization reduce burnout and are strongly associated with high SAQ agreement rates (58).

## 5 Conclusion

This study was the first to investigate the levels of burnout, SOC, and safety attitudes among nurses after COVID-19 in Chinese FSHs, analyzing the associated factors. The research results have important reference value for improving the nursing quality and patient safety management of frontline nurses who have experienced FSH during their return to work. This study suggests that managers should consider the importance of recognising stress, working conditions and teamworking climate in FSHs, and take effective measures to improve nurses’ safety attitudes, such as improving personal accomplishments and SOC, and reducing the degree of depersonalization and working time. A supportive work safety environment plays an important role in reducing job burnout and improving SOC (77). This study also showed that the Chinese version of the SAQ has good consistency within the FSHs and provides baseline data for future studies on patient safety in FSHs. More studies can be combined and analyzed in the future to effectively improve healthcare job safety attitudes and patient safety.

Occupational stress is a key factor that has a negative impact on safety and productivity. Chinese managers usually prefer to solve problems through brainstorming, empowerment, and democracy, which means that the resolution of many problems depends on the wisdom of the majority of employees. This helps to find a balance between employees and conflicts in the process of problem-solving. Management personnel and employees jointly inspect safety hazards and obtain sufficient resources and support, which can influence employees to form a positive attitude towards patients and improve the quality of nursing safety. The management has taken a series of measures to create a good team cooperation atmosphere, optimize work processes, systematize stress recognition education and knowledge training, scientifically set up shifts, establish collaboration models, focus on key populations, thereby improving the work safety environment and strengthening patient safety practices. Consistency plays a crucial role in nursing practice, especially in the quality of interaction between nursing and patients. The interaction between nurses and patients is not only related to the psychological health and quality of life of patients, but is also closely related to the professional satisfaction and work engagement of nurses. Future research will explore the intrinsic mechanisms of nurse consistency and patient interaction quality, providing theoretical support for improving nursing practice.

## 6 Limitations

This study had some limitations. The data sources were all from the Anhui Fangcang shelter hospital in Shanghai, meaning that the results were not representative of nurses in all FSHs. Although the research subjects strictly follow the inclusion and exclusion criteria, there may be a phenomenon of low levels of burnout among some survey subjects, resulting in certain sample selection biases. In addition, survey subjects may have various measurement errors of self-reports, such as recall and social expectation biases, which may limit the generalizability of research results. This study is a cross-sectional design, and there may be related causal confusion and reverse causal relationships. In future studies, managers should obtain informed consent to conduct studies using more FSHs. Future cohort studies could be conducted with continuous attention to the psychological status and work safety attitudes of healthcare workers in FSHs to more accurately analyze associated factors.

## Data Availability

The original contributions presented in the study are included in the article/supplementary material, further inquiries can be directed to the corresponding author.

## Footnotes

^1^ https://www.wjx.cn/
